# Maquiberry Cystatins: Recombinant Expression, Characterization, and Use to Protect Tooth Dentin and Enamel

**DOI:** 10.3390/biomedicines11051360

**Published:** 2023-05-04

**Authors:** Eduardo Pereira de Souza, Milene Ferro, Vinicius Taioqui Pelá, Thais Fernanda-Carlos, Cecília Guimarães Giannico Borges, Even Akemi Taira, Talita Mendes Oliveira Ventura, Ariel Domingo Arencibia, Marília Afonso Rabelo Buzalaf, Flávio Henrique-Silva

**Affiliations:** 1 Department of Genetics and Evolution, Federal University of São Carlos (UFSCar), São Carlos 13565-905, SP, Brazil; edupsouza96@gmail.com (E.P.d.S.); thaisfernandacarlos@gmail.com (T.F.-C.); 2 Department of General and Applied Biology, Institute of Biosciences, São Paulo State University (UNESP), Rio Claro 13506-900, SP, Brazil; milenef@gmail.com; 3 Department of Biological Sciences, Bauru School of Dentistry, University of São Paulo (USP), Bauru 17012-901, SP, Brazil; viniciuspela@gmail.com (V.T.P.); ceciliaguimaraes@usp.br (C.G.G.B.); talitaventura@usp.br (T.M.O.V.); mbuzalaf@fob.usp.br (M.A.R.B.); 4 Center of Biotechnology in Natural Resources, Faculty of Agrarian and Forestry Sciences, Catholic University of Maule (UCM), Talca 3466706, Chile; arieldarencibia@gmail.com

**Keywords:** *Aristotelia chilensis*, phytocystatin, cysteine protease inhibitor, cathepsin, cysteine protease, dentistry, dental erosion

## Abstract

Phytocystatins are proteinaceous competitive inhibitors of cysteine peptidases involved in physiological and defensive roles in plants. Their application as potential therapeutics for human disorders has been suggested, and the hunt for novel cystatin variants in different plants, such as maqui (*Aristotelia chilensis*), is pertinent. Being an understudied species, the biotechnological potential of maqui proteins is little understood. In the present study, we constructed a transcriptome of maqui plantlets using next-generation sequencing, in which we found six cystatin sequences. Five of them were cloned and recombinantly expressed. Inhibition assays were performed against papain and human cathepsins B and L. Maquicystatins can inhibit the proteases in nanomolar order, except MaquiCPIs 4 and 5, which inhibit cathepsin B in micromolar order. This suggests maquicystatins’ potential use for treating human diseases. In addition, since we previously demonstrated the efficacy of a sugarcane-derived cystatin to protect dental enamel, we tested the ability of MaquiCPI-3 to protect both dentin and enamel. Both were protected by this protein (by One-way ANOVA and Tukey’s Multiple Comparisons Test, *p* < 0.05), suggesting its potential usage in dental products.

## 1. Introduction

The cystatin superfamily of proteins is composed of reversible inhibitors of cysteine proteinases, mainly from the family C1A. They present a competitive mechanism, acting as a pseudo-substrate [1,2,3]. Its high inhibitory capacity is due to a tight-binding interaction caused by three conserved contact points between the proteases and the inhibitor. They consist of an N-terminal region containing a glycine residue, a central β-hairpin loop with a Gln-X-Val-X-Gly motif and a C-terminal β-hairpin loop featuring a tryptophan residue [4,5,6]. The cystatin superfamily is subdivided into four families. Three of them—the stefins, the cystatins and the kininogens—are from animal origin, and the last one, the phytocystatins, is present in plants [1,6,7].

Compared to animal cystatins, phytocystatins show high homology with members of the cystatin family, although they lack disulfide bonds as stefins. They also present an additional conserved motif, with an unknown function, in an N-terminal α-helix with the following sequence: [LVI]-[AGT]-[RKE]-[FY]-[AS]-[VI]-X-[EDQV]-[HYFQ]-N [7]. Some phytocystatins have a C-terminal extension containing an SNSL motif, which gives them the capacity to inhibit C13 cys-proteases [8].

Plant cystatins are thought to play a number of physiological roles, such as regulating endogenous cysteine peptidases during seed development and germination [9], senescence [10] and abiotic stresses, such as drought [11], cold [12] and high salinity [13]. The defensive roles of these inhibitors against insects [14], fungi [15] and nematodes [16] have also been reported. This evidence demonstrates how cystatins might be important in the development of biotechnological approaches in agriculture [17].

Phytocystatins have also been described with potential biotechnological applications in medicine, and this is strongly related to their capacity to inhibit human cathepsins [18,19]. These inhibitors have been reported to regulate distinct pathologies, showing anticancer, anti-inflammatory and osteogenic effects [20,21,22]. They are also associated with the inhibition of pathogens of different natures, such as the malaria parasite, *Plasmodium falciparum* [23], and the yeast, *Candida* spp. [24]. Recently, plant cystatins have demonstrated potential applications in dentistry. Studies with a sugarcane cystatin (CaneCPI-5) revealed that this protein decreased the initial erosion of the dental enamel and also reduced biofilm activity and mineral loss related to caries progression [25,26,27].

Maqui (*Aristotelia chilensis* (Mol.) Stuntz) is a native plant of the Elaeocarpaceae family. As a shrubby and perennial species, it grows wild in the central and southern regions of Chile and Argentina. It produces small purple-colored berries, which are typically consumed fresh or as jams and beverages [28,29,30]. These fruits, as well as maqui leaves, have been part of traditional medicine as treatments for sore throats, kidney pain, digestive ailments, fever and scarring injuries [31,32].

Nowadays, maqui has been highlighted due to its phytochemicals, mainly anthocyanins, which are available in high quantities. This fact not only calls attention to its uses as a natural pigment but also to its potential as a health agent. The fruit’s high anthocyanin content has been described as responsible for its high antioxidant properties, which has made maqui to be considered a superfruit [33,34]. In this context, maqui anthocyanins have been reported to act as cardioprotective [35], antidiabetic [36] and anti-inflammatory [37] effectors. Currently, several studies are focused on the addition of plant polyphenols in dental materials, as well as their direct application on the tooth surface [38]. In the context of erosive tooth wear, the use of proteins, polyphenols and natural extracts has demonstrated protection for the tooth structure when applied directly to this surface or through modification of the acquired pellicle [25,39,40]. Although maqui has been studied in terms of its polyphenols, little is known about its genes and proteins.

In this study, we report the identification and phylogenetic analysis of *Aristotelia chilensis* cystatins, as well as the cloning, recombinant expression and inhibitory profile of five distinct proteins. In vitro assays were performed with maquicystatins against papain and human cathepsins L and B, and considerable inhibition was observed. Our results contribute to describing these inhibitors and presenting their interaction with relevant cysteine proteases, reflecting their potential uses in agriculture and medicine. Furthermore, considering cystatin’s applicability in dentistry, MaquiCPI-3, which has the highest production yield, was studied regarding dental enamel and dentin protection capacity. The following null hypothesis was tested: solutions containing different concentrations of MaquiCPI-3 do not protect enamel and dentin against initial erosion in vitro.

## 2. Materials and Methods

### 2.1. RNA Extraction

Wild maqui plants (*Aristotelia chilensis* (Mol.) Stuntz) were selected from the surroundings of the locality of Vilches (35°35′59″ south; 71°11′6″ west), Maule Region, Chile. Plantlets were cultivated in Temporary Immersion Bioreactor (TIB), as described [41]. Bioreactors were maintained at 25 ± 2 °C under natural sunlight and cool-white fluorescent tubes at a light intensity of 100 μM m^−2^ s^−1^. Culture medium (200 mL) was MS basal salts, TDZ 1 mg/L, pH 5.6. Air was enriched with 0.4 MPa CO_2_, while the immersion frequency was every 6 h for 4 min. After three weeks of plant multiplication, RNA extraction (about 1 g biomass) was made from 15-day-old plants using Trizol (Invitrogen, Carlsbad, CA, USA), according to manufacturer instructions. RNA integrity was analyzed by the rRNA pattern in 1% agarose gel, while the purity was checked in a NanoDrop 1000 (Thermo Scientific Inc., Wilmington, DE, USA).

### 2.2. Preprocessing Reads, De Novo Assembly and Completeness Assembly

Transcriptome was sequenced using Illumina HiSeq platform with TruSeq Small RNA Library Preparation Kit. The raw read control quality was verified by FastQC v0.11.5 (http://www.bioinformatics.babraham.ac.uk/projects/fastqc/ (accessed on 4 April 2020)). We found no adapter or primer sequences and trimmed out low-quality raw reads (Phred Q score < 30) using SeqyClean v1.10.09 [42]. Fastx_trimmer v0.0.14 (http://hannonlab.cshl.edu/fastx_toolkit/ (accessed on 4 April 2020)) was used to remove the first 15bp in order to solve the GC bias problem [43,44]. To increase the efficiency of assembly reads, we performed in silico normalization according to Brown and colleagues’ protocol (2012) [45] and de novo assembly using Trinity v2.3.2 [46] with default parameters, generating a reference transcriptome. We evaluated the transcriptome completeness using Bench-marking Universal Single-Copy Orthologs (BUSCO) v4.0.4 [47,48]. The reference transcriptome was compared with a Viridiplantae OrthoDB v10 database, considering a cut-off of 1E--3. The contigs were classified as “complete, single copy”, “complete, duplicated copy”, “fragmented”, or “missing”, depending on the length of the aligned sequence.

### 2.3. Screening for Maquicystatins

Fourteen phytocystatin sequences were used as baits in a local BLASTP procedure: *Fragaria chiloensis* (AWO72930.1), *Prunus dulcis* (BBN70259.1), *Musa acuminata malaccensis* (XP_009387215.1, XP_009386948.1), *Solanum lycopersicum* (AAF23126.1), *Vitis cinerea* var. helleri x *Vitis riparia* (ADD51191.1), *Rosa chinensis* (XP_024185425.1), *Malus prunifolia* (AHZ92270.1), *Camellia sinensis* (ACN85344.1), *Cucumis melo* var. makuwa (KAA0040524.1), *Morus notabilis* (EXC30697.1), *Capsicum annuum* (PHT93956.1) and *Fragaria vesca* subsp. *vesca* (XP_011468407.1, XM_011463703.1). Some criteria were employed to identify the target sequences, such as the existence of initiation and termination codons and, also, the presence of the three typical cystatin motifs: Gly-Gly N-terminal residues, QxVxG in the central loop and P-W C-terminal residues [6,49].

### 2.4. Protein Sequence Analysis

Six different sequences selected according to item 2.3 were named MaquiCPI-1, MaquiCPI-2, MaquiCPI-3, MaquiCPI-4, MaquiCPI-5 and MaquiCPI-6. Sequences were deposited in GenBank (MaquiCPI-1: OQ787102, MaquiCPI-2: OQ787103, MaquiCPI-3: OQ787104, MaquiCPI-4: OQ787105, MaquiCPI-5: OQ787106, MaquiCPI-6: OQ787107). The presence of signal peptides was evaluated with PrediSi software [50] and features such as molecular weight and pI were evaluated by Expasy’s Compute MW/pI software [51]. The maquicystatin amino acid sequences were submitted to a multiple alignment using the Muscle method [52]. This alignment was used to generate a phylogenetic tree. The substitution model inference was determined in the Mega11 platform [53], and the Whelan and Goldman method (WAG) [54] with a discrete gamma distribution with two categories was the most appropriate and used to build a Maximum Likelihood phylogenetic tree. The initial tree was built using the neighbor-joining method, followed by bootstrapping with 1000 pseudo-replicates to calculate the node support values. The final tree was rendered in Mega11 [53], with branches drawn as their substitution distances. Another alignment was performed with MaquiCPIs sequences along with cystatins from *Oriza sativa* ssp. *japonica* (OC-I—P09229-2, OC-II—P20907, OC-III—Q6I570, OC-IV—Q5N806, OC-V—Q0JGM8, OC-VI—Q10Q46, OC-VII—Q10Q47, OC-VIII—Q10J94, OC-IX—Q10Q48, OC-X—P0C579, OC-XI—Q6K309, OC-XII—Q0JNR2; codes are from Uniprot) using the Muscle method [52]. The phylogenetic analysis was performed as described above.

### 2.5. Expression and Purification of Recombinant Cystatins

Total RNA was used as template for cDNA synthesis performed by the High-Capacity cDNA Reverse Transcription Kit (Applied Biosystems, Carlsbad, CA, USA), following manufacturer instructions. The coding regions for *A. chilensis* mature cystatins (MaquiCPIs 1 to 5) were obtained by amplification of the cDNA template using the primers described in Table S1. Forward and reverse primers contained a site for the restriction enzymes *Nde*I and *Sal*I, respectively, for directional cloning into pET28a (Novagen), previously digested with the same enzymes. Briefly, PCRs were performed in a 25 μL reaction containing 200 μM dNTPs (Invitrogen), 1x reaction buffer, 12.5 pmol of each primer, 2.5 U of Taq High Fidelity Pol (Cellco Biotec, Brazil) and the template cDNA. The reaction was carried out with the following cycle: 95 °C for 3 min, followed by 35 cycles at 95 °C for 1 min, 45–55 °C for 1 min and 72 °C for 1 min with final extension at 72 °C for 7 min. The PCR product was digested with *Nde*I and *Sal*I and cloned into pET28a (Novagen) in frame with a 5′ His-tag coding sequence.

Recombinant expression and purification of maquicystatins were performed as previously described [55]. In short, the recombinant plasmids were transformed into *E. coli* Rosetta™️ DE3 competent cells (Novagen), and bacterial cultures were grown at 37 °C and 250 rpm until they reached OD_600_ = 0.5. Expression was induced by the addition of IPTG (isopropyl-β-D-thiogalactopyranoside) to a final concentration of 0.4 mM and performed for 4 h. Due to the presence of a His-tag, the recombinant proteins were purified using Ni-NTA Superflow Resin (Qiagen, Valencia, CA, USA) following the manufacturer’s instructions. All the protein production steps were analyzed by SDS-PAGE 15% as described elsewhere [56]. Purified fractions were dialyzed in PBS buffer (137 mM NaCl, 2.7 mM KCl, 10 mM Na_2_HPO_4_, 1.8 mM KH_2_PO_4_) pH 7.4 (MaquiCPIs 1, 2 and 5) or pH 8 (MaquiCPIs 3 and 4). Total protein quantification was performed using Pierce BCA Protein Assay Kit (Thermo Scientific, Rockford, IL, USA).

### 2.6. Enzyme Inhibition Assays

The inhibitory potential of MaquiCPIs was evaluated against papain (10 nM), human cathepsins B (1.9 nM) and L (3.5 nM) (Calbiochem, San Diego, CA, USA). Enzymes were preincubated in the activation buffer (0.1 M sodium acetate buffer, pH 5.5, containing 2.5 mM dithiothreitol (USB) in a final volume of 500 µL) for 5 min at 37 °C. The fluorogenic substrate Z-Phe-Arg-AMC (2.5 µM, 8 µM and 20 µM for papain, cathepsin L and B, respectively) (Calbiochem) was used to determine the catalytic activity of the cys-proteases. Cystatins were added to the reactions at increasing concentrations. Fluorescence changes were monitored continuously using Hitachi F-2500 spectrofluorometer (Hitachi) at λ_ex_ = 380 nm and λ_em_ = 460 nm. The inhibitory potential of maquicystatins was determined using the residual enzymatic activity of the cys-proteases after the addition of the inhibitor. Slope values were obtained using the FL Solutions 2.0 program, and the apparent inhibition constant (Ki_app_) values were obtained using the following equation [57]:

V_0_/V_i_ = 1 + [I]/Ki_app_, (1)

where V_0_ and V_i_ are the velocities of substrate hydrolysis in the absence and presence of different inhibitor concentrations, [I], respectively. The assays were performed in triplicate, and the Ki parameters were obtained from the following equation [57]:

Ki = Ki_app_/(1 + [S]/Km). (2)

The following Km values were used to correct the values for substrate competition: 23 μM cathepsin B, 2 µM cathepsin L and 10 µM papain [58].

### 2.7. Definition of MaquiCPI-3 Concentrations for the Prevention of Enamel and Dentin Erosion In Vitro

#### 2.7.1. Preparation of Bovine Enamel and Dentin Samples

One hundred and forty samples of bovine enamel and root dentin were prepared (4 mm × 4 mm × 3 mm) using a cutting machine (ISOMET Low-Speed Saw Buehler, Lake Bluff, IL, USA). All samples were polished with 300, 600 and 1200 granulation silicon carbide sandpaper (Extec Corp. Papers, Buehler, Lake Bluff, IL, USA). Subsequently, a felt was used (Polishing cloth, Buehler, Lake Bluff, IL, USA) with diamond solution (Extec Corp., Buehler, Lake Bluff, IL, USA). A total of 120 μm of tooth structure (enamel or dentin) was removed. The samples were washed in an ultrasonication bath (5 min) after each granulation, as described above, and stored at 4 °C until the beginning of the experimental procedure [59].

#### 2.7.2. Human Saliva Acquisition

Nine adults (four men and five women) aged approximately 30 years were selected for this study based on the following selection criteria for general health: non-pregnant women, non-smokers, no use of prolonged medication and without systemic diseases. They were also checked for the following oral health-specific criteria: without active caries or periodontal disease and presenting normal salivary flow (stimulated saliva >1.0 mL/min and unstimulated saliva >0.3 mL/min) [27]. The stimulated saliva was collected in the morning period (from 9:00 to 9:30), using paraffin wax for 10 min. Then, all saliva was pooled, and the supernatants were separated using centrifugation (14,000× *g*, 20 min, at 4 °C). Finally, the saliva was aliquoted and stored at −80 °C [25].

#### 2.7.3. Treatment Groups

The samples (enamel and dentin) were randomly distributed to 7 groups (*n* = 20/group): (1) deionized water, negative control (Control); (2) commercial solution—Elmex^®^, composed of 800 ppm Sn^+2^, 500 ppm F (SnCl_2_/NaF/AmF), pH 4.43, positive control, GABA International AG, Therwil, BL, Switzerland (Elmex); (3) 0.1 mg/mL CaneCPI-5, pH 7.88 (CaneCPI-5) [25]; (4) 0.1 mg/mL MaquiCPI-3 (0.1 MaquiCPI-3); (5) 0.25 mg/mL MaquiCPI-3 (0.25 MaquiCPI-3); (6) 0.5 mg/mL MaquiCPI-3 (0.5 MaquiCPI-3); and (7) 1.0 mg/mL MaquiCPI-3 (1.0 MaquiCPI-3). The pH of the treatments containing MaquiCPI-3 ranged between 7.10 and 7.30.

#### 2.7.4. Treatment, Acquired Pellicle Formation and Erosive Process

At first, the surfaces of the enamel and dentin samples were individually treated (250 μL), according to the groups displayed above for 2 h, at 37 °C, under agitation of 300 rpm. After this period, the samples were washed (10 s) and dried (5 s). Then, the acquired pellicle was individually formed by exposure to 250 μL of human saliva for 2 h, at 37 °C, under agitation of 300 rpm. Subsequently, the samples were washed (10 s) and dried (5 s). Lastly, the erosive process was also individually performed by incubating the samples in 1 mL of 1% citric acid (pH = 3.6) for 1 min, at 25 °C, under agitation of 300 rpm. Again, the samples were washed (10 s) and dried (5 s). These experimental procedures were repeated 3 times, on 3 consecutive days. Between the days of the experiment intervals, the samples were stored under humidity control at 4 °C [25].

#### 2.7.5. Percentage of Surface Microhardness Change (%SMC)

The values were obtained with a Microhardness Tester, using a Knoop diamond (SMH—HMV-2000, Shimadzu, Kyoto, Japan). Six indentations per sample were made (at intervals of 25 µm between them) on a defect-free surface area at the beginning of the experiment (SM _baseline_) and after the experimental procedure (SM _final_). For the enamel samples, a load of 50 g and a dwell time of 15 s was used [25], while for the dentin samples, a load of 10 g and a dwell time of 15 s was used [60]. The data were tabulated as percentage of surface microhardness change (%SMC), according to the following equation [25]:

%SMC = ([SM_baseline_—SM_final_]/SM_baseline_) × 100. (3)

#### 2.7.6. Statistical Analysis

GraphPad Prism software (version 6.0 for Windows, GraphPad Software Inc., La Jolla, CA, USA) was used. All analyses were checked for normality (Kolmogorov–Smirnov test) and homogeneity (Bartlett test). The data from enamel and dentin surfaces were analyzed using One-way ANOVA and Tukey’s Multiple Comparisons Test. The significance level was set at 0.05.

## 3. Results

### 3.1. In Silico Analysis of Cystatin Sequences

**Figure 1:**
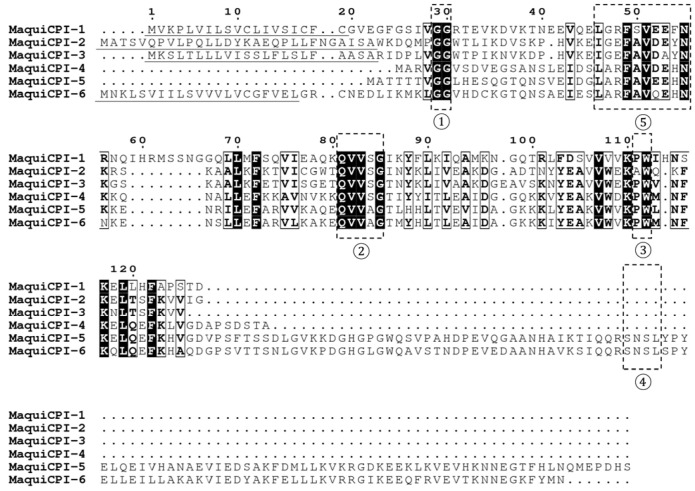
Multiple alignment analysis of maquicystatins amino acid sequences. Regions highlighted by dashed boxes indicate the papain-like inhibitory motifs ①: GG, ②: QxVxG and ③: PW, the legumain-like inhibitory motif ④: SNSL and the exclusive phytocystatins motif ⑤: [LVI]—[AGT]—[RKE]—[FY]—[AS]—[VI]—X—[EDQV]—[HYFQ]—N. Signal peptides from each sequence are underlined in black. Alignment was generated using Muscle software with default parameters.

We identified six putative non-redundant cystatin sequences (named MaquiCPI-1 to 6) in an *Aristotelia chilensis* transcriptome of plantlets cultivated in TIBs (Table S2). Figure 1 shows the multiple alignments between MaquiCPI amino acid sequences, highlighting the cystatin inhibitory motifs and the characteristic N-terminal plant cystatin motif. All of them present the typical cystatin motifs (GG, QxVxG and W), while MaquiCPI-5 and MaquiCPI-6 have a C-terminal extension, described as a cystatin-like domain, which includes an additional SNSL legumain inhibitory motif. All proteins, except MaquiCPI-4 and MaquiCPI-5, possess a putative signal peptide sequence of about 19-28 amino acids, and they comprise proteins with 101 to 224 amino acid residues. The molecular weight ranges from 11 to 25 kDa.

In all protein sequences, the GG dipeptide mot if is conserved in the N-terminal region. The typical phytocystatin motif in *A. chilensis* is presented in all groups as [LI]-[AG]-[RE]-F-[AS]-V-[EDQ]-[EA]-[HYF]-N. The central loop motif (QxVxG) diverged between proteins with and without C-terminal extension, displaying the QVVAG sequence for the former and QVVSG for the latter. The dipeptide motif from the C-terminal loop (PW) is conserved in most cystatins, although MaquiCPI-2 has an alanine residue instead of a proline preceding the tryptophan.

**Figure 2:**
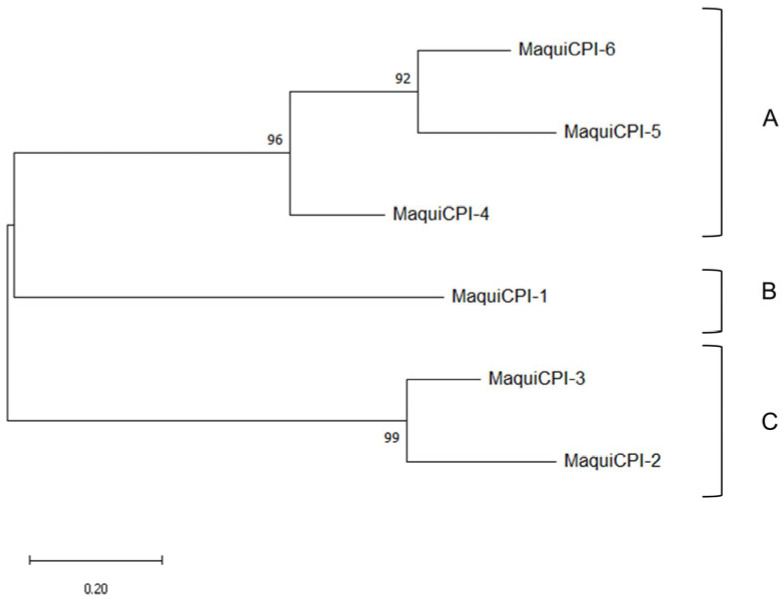
*Aristotelia chilensis* phytocystatins phylogenetic tree. A, B and C correspond to different phylogenetic groups. Evolutionary analysis by Maximum Likelihood method using the Whelan and Goldman model [54]. A discrete Gamma distribution was used to model evolutionary rate differences among sites (2 categories). The tree is drawn to scale, with branch lengths measured in the number of substitutions per site. Nodes support values are shown next to the branches. This analysis involved 18 amino acid sequences, where only positions with 75% site coverage or higher were kept.

**Figure 3:**
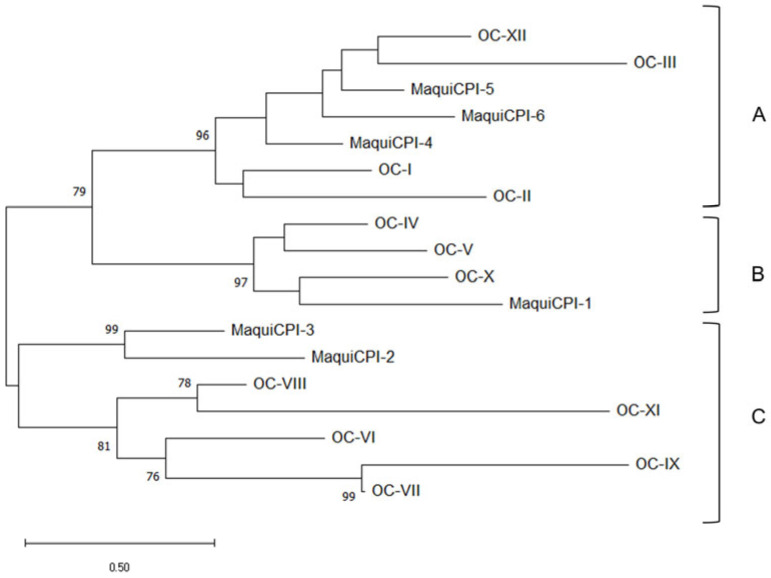
*Aristotelia chilensis* and *Oriza sativa* spp. *japonica* phytocystatins phylogenetic tree. A, B and C correspond to different phylogenetic groups. Evolutionary analysis by Maximum Likelihood method using the Whelan and Goldman model [54]. A discrete Gamma distribution was used to model evolutionary rate differences among sites (2 categories). The tree is drawn to scale, with branch lengths measured in the number of substitutions per site. Nodes support values are shown next to the branches. This analysis involved 18 amino acid sequences, where only positions with 75% site coverage or higher were kept.

A phylogenetic tree was built including the six identified cystatins from *A. chilensis* (Figure 2). The amino acid sequences were clustered into three groups. Group A is composed of MaquiCPI-4 and an inner clade, formed by the extended cystatins MaquiCPI-5 and MaquiCPI-6. The proteins of this group share a highly conserved N-terminal α-helix motif: LARFAV-[DEQ]-EHN. MaquiCPI-1 is the only example from group B, and it is characterized by an extension of eight amino acid residues starting in position 59 from the alignment (Figure 1). Finally, group C comprises MaquiCPI-2 and MaquiCPI-3, which lack any extension and the N-terminal α-helix displays the IGEFAVD-[EA]-YN pattern. When a phylogenetic tree (Figure 3) was constructed using cystatins from maqui and *Oriza sativa* ssp *japonica*, three clusters were also observed. MaquiCPIs were distributed within the groups following the same pattern described in Figure 2.

### 3.2. Protein Expression Purification

**Figure 4:**
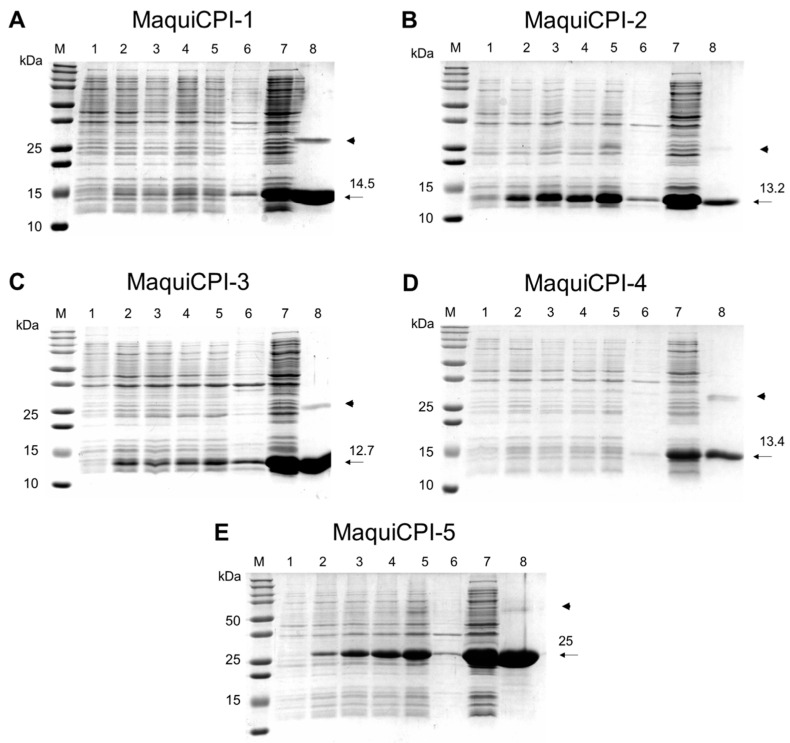
Analysis of recombinant expression and purification of maquicystatins. (**A**) MaquiCPI-1. (**B**) MaquiCPI-2. (**C**) MaquiCPI-3. (**D**) MaquiCPI-4. (**E**) MaquiCPI-5. SDS-PAGE (15%) showing in M: Precision Plus Protein Standards—Bio-Rad; 1: cell extract before induction (0 h); 2, 3, 4 and 5: cell extract after 1, 2, 3 and 4 h induction with IPTG; 6 and 7: insoluble and soluble fractions after cell lysis; 8: purified recombinant protein. The arrows indicate the recombinant protein with its respective theoretical molecular mass. The arrowheads indicate bands with double the predicted size.

The ORFs from five of six cystatins (MaquiCPI-1 to MaquiCPI-5) were successfully obtained by PCR using specific primers and subcloned in a pET28a vector in frame with an N-terminal His-tag coding sequence. Expression was induced in *E. coli* Rosetta (DE3), and purification was successfully performed in a single step using affinity chromatography. SDS PAGE analysis (Figure 4) revealed that, after sonication, the recombinant proteins were mostly present in the soluble fraction and were able to be directly purified. The yields of purified MaquiCPIs 1 to 5 were 17.5, 23.6, 120.0, 18.5 and 44.6 mg per liter of cell culture, respectively. The amounts of pure protein were sufficient for performing activity assays.

### 3.3. Inhibitory Activity Assay

MaquiCPIs were tested against papain (Figure S1) and human cathepsins B (Figure S2) and L (Figure S3) to assess their inhibition profile. The Ki of each *A. chilensis* cystatin tested against the cys-proteases are presented in Table 1.

### 3.4. Percentage of Surface Microhardness Change on Enamel

**Figure 5:**
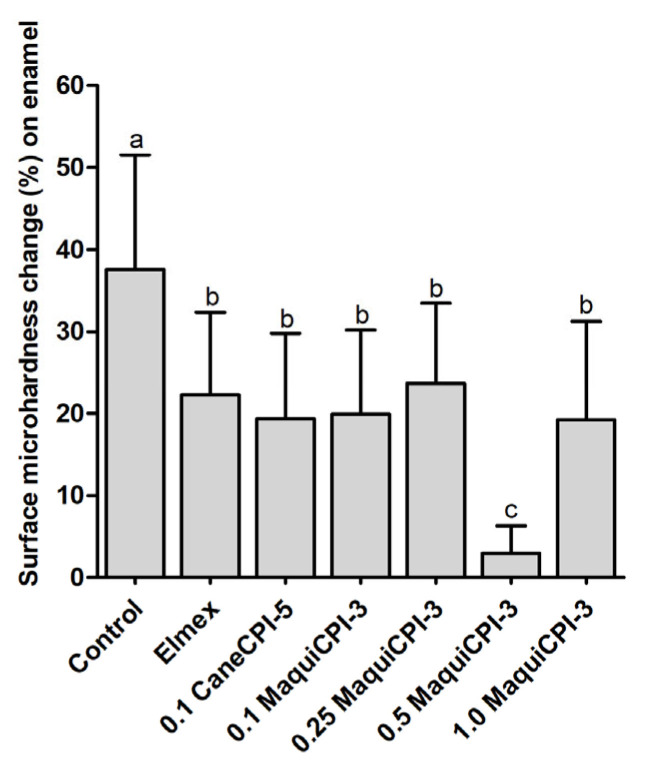
Percentage of surface microhardness change (%SMC) according to the different treatments on enamel: Control, Elmex, 0.1 mg/mL CaneCPI-5, 0.1 mg/mL MaquiCPI-3, 0.25 mg/mL MaquiCPI-3, 0.5 mg/mL MaquiCPI-3 and 1.0 mg/mL MaquiCPI-3. Smaller bars mean less erosion. Different letters mean significant differences between treatments, and error bars indicate standard deviations. One-way ANOVA and Tukey’s Multiple Comparisons Test (*p* < 0.05). *n* = 20.

The %SMC results on the enamel surface show that the Control group presented a significantly greater surface microhardness change (37.56% ± 13.98) compared to the other treatments (*p* < 0.05), which show no significant differences between Elmex (22.30% ± 10.06), CaneCPI-5 (19.37% ± 10.41), 0.1 MaquiCPI-3 (19.93% ± 10.26), 0.25 MaquiCPI-3 (23.72% ± 9.74) and 1.0 MaquiCPI-3 (19.23% ± 12.01) (*p* < 0.05). In addition, the 0.50 MaquiCPI-3 treatment demonstrated the best protective effect against initial dental erosion (significantly), with the least surface microhardness change (2.90% ± 3.35) compared to all the other treatments (*p* < 0.05) (Figure 5).

### 3.5. Percentage of Surface Microhardness Change on Dentin

**Figure 6:**
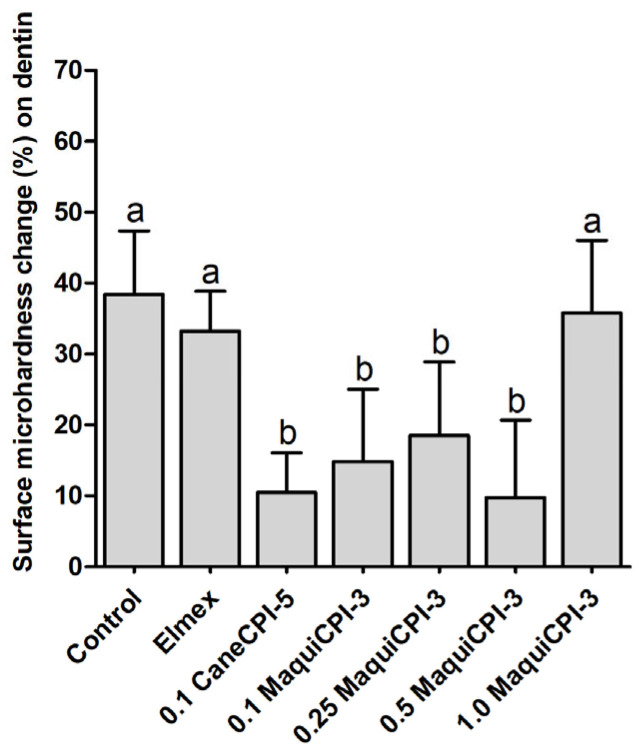
Percentage of surface microhardness change (%SMC) according to the different treatments on dentin: Control, Elmex, 0.1 mg/mL CaneCPI-5, 0.1 mg/mL MaquiCPI-3, 0.25 mg/mL MaquiCPI-3, 0.5 mg/mL MaquiCPI-3 and 1.0 mg/mL MaquiCPI-3. Smaller bars mean less erosion. Different letters mean significant differences between treatments, and error bars indicate standard deviations. One-way ANOVA and Tukey’s Multiple Comparisons Test (*p* < 0.05). *n* = 20.

The %SMC results on dentin surface show that the Control, Elmex and 1.0 MaquiCPI-3 groups presented significantly greater surface microhardness change in comparison to the other treatments (*p* < 0.05), without a significant difference between each other (38.38% ± 8.97, 33.25% ± 5.62 and 35.77% ± 10.24, respectively). The treatments CaneCPI-5 (10.49% ± 5.57), 0.1 MaquiCPI-3 (14.87% ± 10.18), 0.25 MaquiCPI-3 (18.52% ± 10.33) and 0.50 MaquiCPI-3 (9.76% ± 10.93), which also did not differ significantly between each other (*p* < 0.05), presented the lowest surface microhardness change (Figure 6).

## 4. Discussion

In this work, we identified six different maquicystatins sequences in a transcriptome of maqui plantlets cultivated in a Temporary Immersion Bioreactor (TIB). Five of them were cloned and recombinantly expressed in *E. coli* Rosetta (DE3) and tested against different cysteine proteases. Additionally, one of them was tested to protect tooth dentin and enamel, a possible application of this protein.

All deduced proteins share three conserved motifs that help them to interact with the active site cleft of cysteine proteases. These are a conserved glycine and a LARFAV motif in the N-terminal region, a very conserved QxVxG motif in a central loop of the protein and a second loop close to the C-terminal region presenting a tryptophan residue [6,49]. MaquiCPI-5 and MaquiCPI-6 have a C-terminal extension containing a motif composed of four amino acids: SNSL, related to the ability to inhibit legumains [8].

The presence of putative signal peptides was also investigated. It was clear that MaquiCPI-1, MaquiCPI-3 and MaquiCPI-6 presented this classic sequence for secretion, suggesting they are extracellular. On the other hand, MaquiCPI-2 did not present a typical signal for secretion. However, after cloning and expression of the entire MaquiCPI-2 sequence, the expression was unsuccessful. This question led us to a deeper investigation of the first residues of the MaquiCPI-2 N-terminal sequence. Another analysis was made using the prediction software iPSORT [61], and this demonstrated, with high probability, that the amino acids from 1 to 30 signalize the cystatin to the chloroplast. Madureira et al. (2006) identified, by immunolocalization, traces of a tomato multicystatin naturally occurring in chloroplasts [62]. Prins et al. (2008) described the effects of a transgenic tobacco plant overexpressing the phytocystatin OC-I, from rice, on leaf senescence. They observed that the cystatin was not only present in vacuoles and cytosol but also in chloroplasts and, compared to the wild-type plant, there were differences in the protein content and turnover, which culminated in senescence delay [63]. Alomrani et al. (2021) investigated the behavior of a transgenic *Arabidopsis thaliana* event expressing OC-I targeted to the chloroplast. They evaluated its effect on photosynthesis and the accumulation of leaf pigments was evident, suggesting a retardation in senescence. This indicates that the cystatin targets in the chloroplasts might be related to pigment degradation and/or biosynthesis [64]. These reports are in accordance with recent research revealing an active cysteine protease (HvPAP14) in barley chloroplasts. The protein is activated in the thylakoid lumen due to its acidic pH, and its active form is found in the thylakoid membranes, where it performs a proteolytic role. It was also observed that HvPAP14 contributes to the degradation of the large subunit of Rubisco [65]. A prior report also described the presence of cysteine proteases in the thylakoid lumen of spinach leaves [66]. Therefore, MaquiCPI-2 might be associated with the maintenance of tissue homeostasis, and a deep investigation about its function and localization should be performed in the future.

It is well known that signal peptides usually negatively interfere with recombinant protein expression [55]. When the MaquiCPI-2 predicted signal sequence was removed, the protein expression was successful. This fact supports the possibility of this N-terminal sequence’s involvement in the localization of this cystatin in the plant cell. To establish the size of the peptide removed, we compared the N-terminal regions of MaquiCPI-2 and MaquiCPI-3. The signal peptide cleavage site prediction of MaquiCPI-3 is between residues 22 and 23 (AASA-RI). The alignment shows a similar region in MaquiCPI-2 (AISA-WK) between residues 29 and 30. Six residues ahead, there is a conserved block among both proteins (GGWT). Accordingly, due to the similarities described and to maintain the probable distances, the first 29 amino acids were discarded in the plasmid construction, keeping the tryptophan residue, which, in the iPSORT prediction, is part of the signal peptide.

A phylogenetic analysis among MaquiCPIs was carried out, and three groups were identified. They share characteristics related to amino acid sequence extensions and the plant cystatin exclusive N-terminus alpha helix motif. The analysis was complemented with amino acid sequences from 12 rice cystatins, which are well characterized. It was observed that, despite the evolutionary distance among the species, only three groups were formed. This supports the evidence of three groups formed by MaquiCPIs, which is what happens in other species such as citrus, barley and turnip, as well as rice [18,67,68,69]. According to Balbinott and Margis (2022), the diversification in clusters occurred due to the evolution of an ancestral cystatin gene from the most recent common ancestor (MRCA) of Viridiplantae [70]. This gene was subjected to an *in tandem* duplication, resulting in a carboxy-extended form, which is present only in plants. This extension underwent a process of neofunctionalization, culminating in the ability to inhibit legumain-like proteases (from the C13 family). In *A. chilensis*, these cystatins are represented by MaquiCPI-5 and MaquiCPI-6. Parallelly, the evolution of the remaining single-domain phytocystatin resulted in part of the current phytocystatins with introns. Genes with the same characteristics also originated from recent losses of the second domain of carboxy-extended phytocystatins. MaquiCPI-4 is an example that presents these attributes. Finally, a single-domain cystatin from the MRCA of flowering plants was retroduplicated. It resulted in intron loss and, consequently, in the emergence of intronless phytocystatins. Duplication and diversification events culminated in two distinct clusters. In maqui plants, one group is represented by MaquiCPI-1, and the other by MaquiCPI-2 and MaquiCPI-3.

Five of the six identified phytocystatins could be cloned and recombinantly expressed with a satisfactory yield, mostly in the soluble fraction. Regarding yield, MaquiCPI-3 stands out among the phytocystatins due to the fact that more than 100 mg can be produced per liter of *E. coli* culture. Although there are divergences in the methods of production, such as culture media, IPTG concentration and *E. coli* strain, it is clear that the quantity of MaquiCPI-3 produced is superior to most phytocystatins produced with this expression system (Table S3). The yield is also greater than that of human cystatins of biotechnological interest, such as stefin B and cystatin C. Therefore, the high production level of these peptidase inhibitors reveals considerable potential for pharmaceutical, agricultural and biotechnological applications in industry.

When we analyzed MaquiCPIs by SDS-PAGE, we noticed additional bands that were twice their molecular weight. Probably, these homodimers are generated by domain swapping, which was also observed in other phytocystatins [71].

All maquicystatins were able to inhibit papain efficiently, with Ki values of 7.13 nM, 1.42 nM, 3.29 nM, 2.99 nM, and 5.05 nM from MaquiCPIs 1 to 5. The inhibitory capacity against papain family peptidases indicates that the recombinant MaquiCPIs were produced in the right conformation and proves that they are effective cysteine peptidase inhibitors.

Cathepsin B was inhibited only by MaquiCPIs 1, 2 and 3 in the nanomolar order, with Ki of 35.74 nM, 20.97 nM and 21.94 nM, respectively. However, MaquiCPIs 4 and 5 showed a lower inhibition potential, with Ki in the micromolar order (0.876 µM and 5.47 µM). Maquicystatins 4 and 5 belong to the same phylogenetic group (Figure 2 and Figure 3). This group is formed by cystatins with or without C-terminal extensions of which the genes present introns [70]. By amplifying the genomic DNA, we observed that the sequence associated with MaquiCPI-4 possesses an intron (data not shown). There is evidence that phytocystatins encoded by genes with introns are less likely to inhibit cathepsin B [70], presenting higher Ki values or no observable inhibition. Sugarcane cystatin genes with introns are represented by CaneCPI-1, CaneCPI-2, CaneCPI-3 and CaneCPI-6. The first and second do not have any C-terminal extension and present Ki values higher than 100 nM. The latter is extended, and the Ki values surpass 1.5 µM [19]. Other cystatins encoded by genes with introns from clementine (CclemCPI-3), sweet orange (CsinCPI-2) [18], amaranth (AhCPI) [72] and barley (HvCPI-4) [73] do not present detectable cathepsin B inhibition.

The difficulty of inhibiting cathepsin B is related to the existence of an occlusion loop in the enzyme that blocks the catalytic cleft, hampering their interaction [74]. When cystatins are able to inhibit cathepsin B, a process of two steps can be observed. First, the occlusion loop undergoes a conformational change, unblocking the active site, then allowing the inhibition process [75,76]. This mechanism is associated with phytocystatins N-terminal region [77,78]. The inhibition of cathepsin B by phytocystatins has revealed their potential to control health issues such as cancer and inflammatory diseases [20,22].

All of the *A. chilensis* cystatins were able to inhibit cathepsin L with satisfactory Ki values of 0.34, 0.33, 0.38, 0.57 and 1.25 nM, from MaquiCPI-1 to 5. These values indicate that all MaquiCPIs have the potential to control pathologies resulting from deregulated function or cellular quantities of this protease. As cathepsin L is associated with neurological problems such as Parkinson’s disease, urological issues such as proteinuria and even cancers [79,80,81], it would be of great relevance to study the intervention of maquicystatins in these disorders. Recently, the inhibition of cathepsin L has received attention as a strategy for controlling the SARS-CoV-2 cycle because it is essential for one of the viral entry pathways into the cell [82].

This study brought important results regarding the use of MaquiCPI-3 in the field of dentistry. Our in vitro protocol was designed to evaluate the protective effect and the best concentration of MaquiCPI-3 against an initial dental erosion process. In this sense, we used bovine enamel and dentin samples, which demonstrate a similar structure to human tooth samples [83]. Additionally, we carried out the erosive challenge with citric acid to simulate an extrinsic erosion process (similar to the consumption of citric juices) [84]. Regarding the sequence of application, we followed our previous protocols of “acquired pellicle engineering”, in which the treatment is used before the acquired pellicle formation [25,85]. Furthermore, we used two treatments as positive controls: Elmex^®^ and CaneCPI-5. The first is a highly effective commercial solution for controlling erosive tooth wear, containing fluoride and tin [86]. The second solution, containing a sugarcane-derived phytocystatin, has also demonstrated similar protection to Elmex^®^ for enamel erosion in different protocols [87,88,89,90]. Although the protocol employed in this in vitro study might have been suitable to answer the questions posed, some limitations might be acknowledged, such as the long treatment time (2 h), which does not reproduce the clinical condition, since mouthwashes are typically used between 1 and 2 min. In addition, the time of formation of the acquired pellicle may have also allowed the denaturation of proteins present in human saliva due to the long incubation time. Another fact that deserves to be pointed out is the intact model of pellicle formation, which does not happen in the oral cavity because of the presence of salivary flow. Despite another phytocystatin (CaneCPI-5) that has been shown to be effective in protecting enamel against erosion in vitro [25,87,88,89], in situ [27,59] and in vivo [85,91], this is the first study evaluating the protective potential of a Maqui-berry-derived cystatin. Our results demonstrate that all MaquiCPI-3 concentrations (ranging from 0.1 to 1.0 mg/mL) were effective in protecting enamel against initial dental erosion. Our group has demonstrated that CaneCPI-5 has a strong binding force to hydroxyapatite and that, similarly to human cystatin-B [91], when present in the AP, is resistant to removal by citric and lactic acids [25]. Thus, we suggest that MaquiCPI-3 binds preferably to the enamel surface due to its high binding force to hydroxyapatite [25] and that, after binding, it remodels the architecture of the whole AP, increasing the amount of acid-resistant proteins within this integument [85]. It is important to highlight that 0.5 mg/mL MaquiCPI-3 showed significantly higher protection when compared to the other concentrations and the positive control groups (Elmex^®^ and CaneCPI-5). This is an important finding since, so far, Elmex^®^ is the commercial product with the best results to protect against erosion. It is based on the combination of three inorganic components (Elmex^®^—SnCl_2_/NaF/AmF) [92,93], while our MaquiCPI-3 solution is based on a single organic component. This good performance may be related to the ideal amount of MaquiCPI-3 to bind to available enamel sites so that there is no lack or excess of this treatment on enamel. In addition, 1.0 mg/mL MaquiCPI-3 demonstrated intermediate protection (similar to the concentrations 0.1 and 0.25 mg/mL), showing that there is no need to test higher concentrations.

When evaluated for protection against dentin erosion, MaquiCPI-3 led to distinct results. This might be explained by the different composition of the dentin tissue. This layer, opposite to enamel, is composed of a large organic content (collagen) that, when demineralized, slows down the progression of erosion. However, this layer can be degraded by matrix metalloproteinases (MMPs) and cysteine cathepsins (CCs) [94], allowing the progression of erosion [95]. As seen in this study, MaquiCPI-3 inhibits CCs, similarly to CaneCPI-5 [25]. Thus, one of the mechanisms by which MaquiCPI-3 (at concentrations ranging from 0.1 to 0.5 mg/mL) protects against dentin erosion might be through the inhibition of CCs. Another mechanism might involve the binding of MaquiCPI-3 to hydroxyapatite on the dentin surface, thus modifying the AP architecture, as mentioned for enamel [25,85]. The highest MaquiCPI-3 concentration (1.0 mg/mL) did not protect the dentin. One probable reason might be protein dimerization through domain swapping (and consequent inactivation) since this is common to other phytocystatins [71]. One unexpected finding of this study was the lack of a protective effect against dentin initial erosion for Elmex^®^. Although this commercial product is effective against enamel and dentin erosion, its protection is usually evidenced in protocols involving more severe erosive and abrasive challenges [87,89,96]. Moreover, dentin has lower mineral content compared to enamel, and the stannous ion is a potent reactant with hydroxyapatite [97]. This, along with the short erosive challenge (3 min), might not have allowed the protective action of Elmex^®^ to occur.

## 5. Conclusions

In summary, we identified six different cystatins from a transcriptome of *Aristotelia chilensis* cultivated in Temporary Immersion Bioreactors. They present the three motifs that form the tripartite wedge as well as the alpha helix phytocystatin exclusive motif. Phylogenetically, they are distributed into three distinct groups, following the same pattern as rice and other species. Recombinantly expressed maquicystatins presented inhibitory activity in nanomolar order against papain and human cathepsins B (except MaquiCPIs 4 and 5) and L. Considering the limitations of an in vitro design of initial erosion, MaquiCPI-3 seems to be a promising agent for inclusion in dental products to protect against enamel (at 0.5 mg/mL) and dentin (at 0.1 mg/mL) erosion. Future studies employing protocols that more closely resemble the clinic, such as in situ design, shorter treatment times and prolonged erosive challenges, also associated with abrasive ones, are necessary to pave the way for the use of MaquiCPI-3 in preventive dentistry.

## Figures and Tables

**Table 1:** Inhibition of papain, cathepsin L, and cathepsin B by MaquiCPIs.

	Inhibition Constant—Ki (nM)		
Inhibitor	Papain	Cathepsin L	Cathepsin B
MaquiCPI-1	7.13 ± 1.30	0.34 ± 0.07	35.74 ± 0.84
MaquiCPI-2	1.42 ± 0.39	0.33 ± 0.07	20.97 ± 3.78
MaquiCPI-3	3.29 ± 0.58	0.38 ± 0.08	21.94 ± 3.01
MaquiCPI-4	2.99 ± 0.34	0.57 ± 0.11	876.70 ± 53.99
MaquiCPI-5	5.05 ± 0.82	1.25 ± 0.25	5470 ± 630

## Data Availability

The raw sequence data from the transcriptome of *A. chilensis* are available in the Short Read Archive (SRA) GenBank database: Bioproject (PRJNA953631), BioSample: (SAMN34121413) and SRA (SRR24111384-SRR24111389). The coding sequences for maquicystatins are available in Genbank with the Accession Numbers from OQ787102 to OQ787107.

## Supplementary Materials

The following supporting information can be downloaded at: https://www.mdpi.com/article/10.3390/biomedicines11051360/s1, Figure S1: Papain (*Carica papaya*) inhibition by recombinant maquicystatins. Figure S2: Human cathepsin B inhibition by recombinant maquicystatins. Figure S3: Human cathepsin L inhibition by recombinant maquicystatins. Table S1: Primer sequences used to amplify maquicystatins coding sequences. Table S2: Sequence characteristics from *Aristotelia chilensis* cystatins. Table S3: Plant cystatin expression yields in *Escherichia coli* (mg per liter of culture). References [15,18,25,72,84,98,99,100,101,102,103] are cited in the Supplementary Materials.
