# Supplementary material for: Maquiberry Cystatins: Recombinant Expression, Characterization, and Use to Protect Tooth Dentin and Enamel
Source: Biomedicines. 2023 May 4;11(5):1360. doi: 10.3390/biomedicines11051360 (PMC10216612; doi:10.3390/biomedicines11051360)
Supplement: Supplementary file 1 [file biomedicines-11-01360-s001.zip › Supplementary Figures - Souza et al. 2023.pdf]

Supplementary Figures

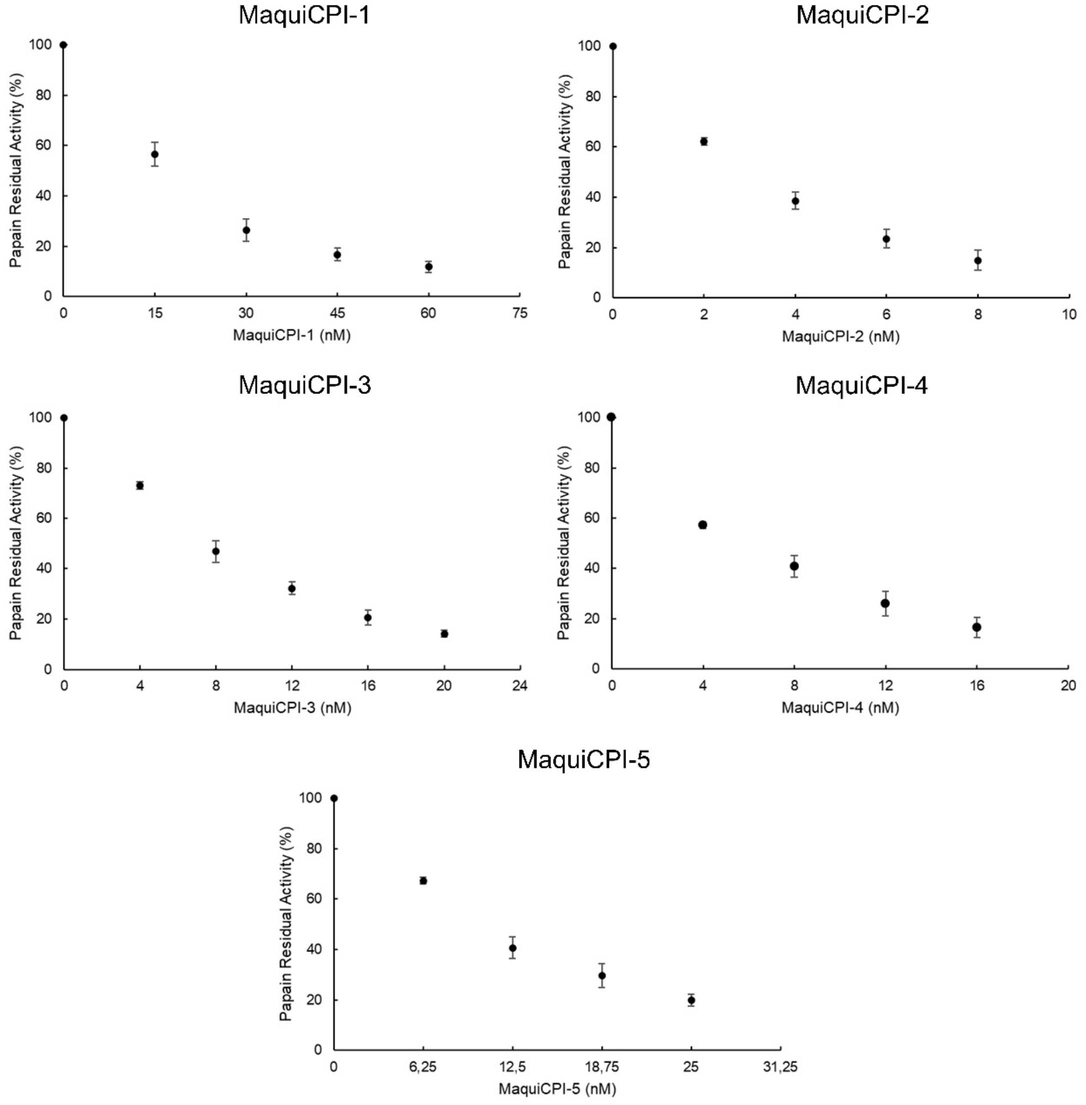

Figure S1: Papain (*Carica papaya*) inhibition by recombinant maquicystatins. The inhibitory activity of maquiCPIs against papain was measured by a fluorimetric assay using Z-FR-MCA as a substrate. The graphics indicate the inhibition kinetics of each maquicystatin, which was obtained with the enzyme residual activity while increasing concentrations of inhibitor were added to the system. The values were indicated as the mean value ± the standard error of three independent experiments.

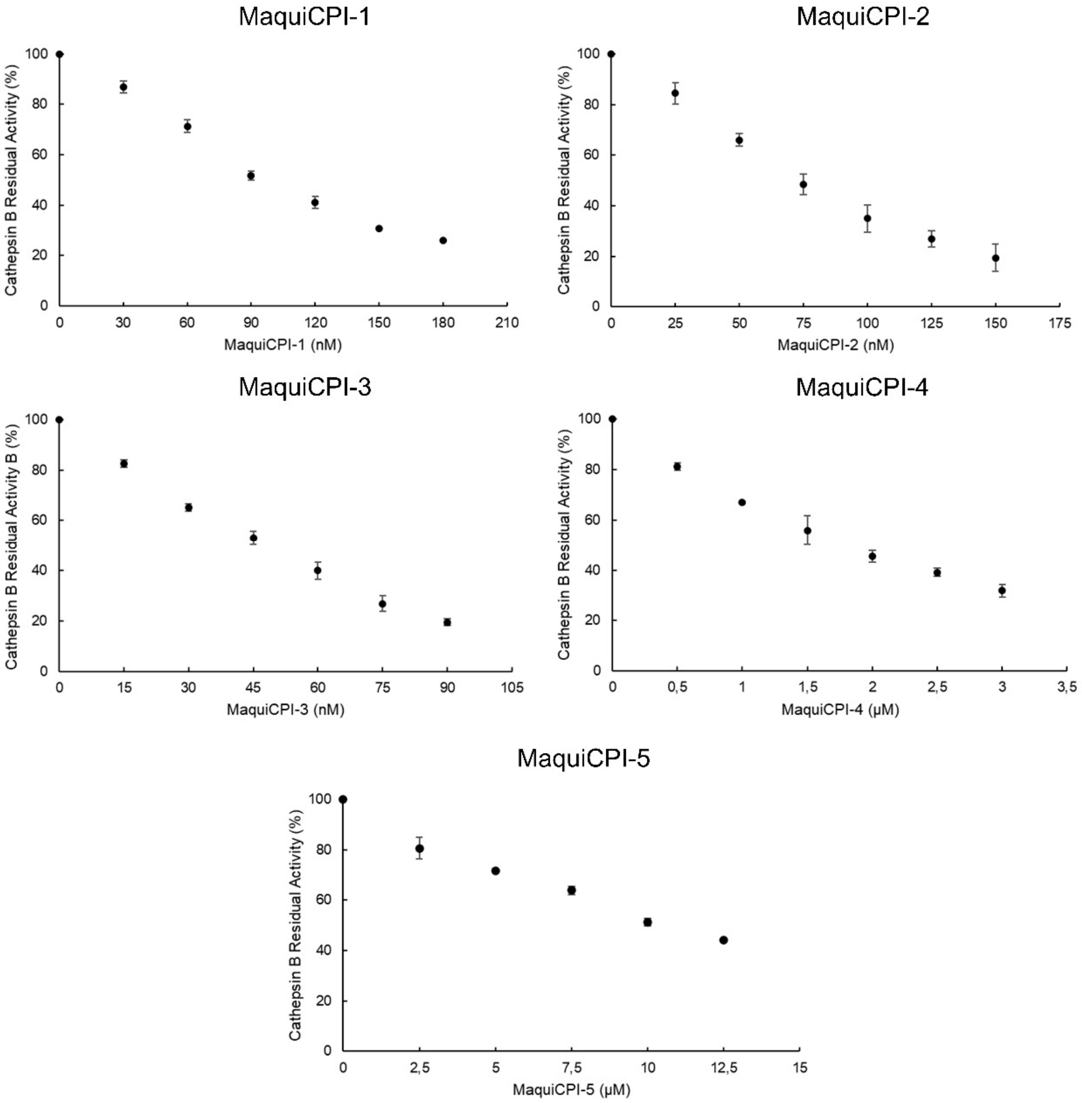

Figure S2: Human Cathepsin B inhibition by recombinant maquicystatins. The inhibitory activity of maquiCPIs against Cathepsin B was measured by a fluorimetric assay using Z-FR-MCA as a substrate. The graphics indicate the inhibition kinetics of each maquicystatin, which was obtained with the enzyme residual activity while increasing concentrations of inhibitor were added to the system. The values were indicated as the mean value ± the standard error of three independent experiments.

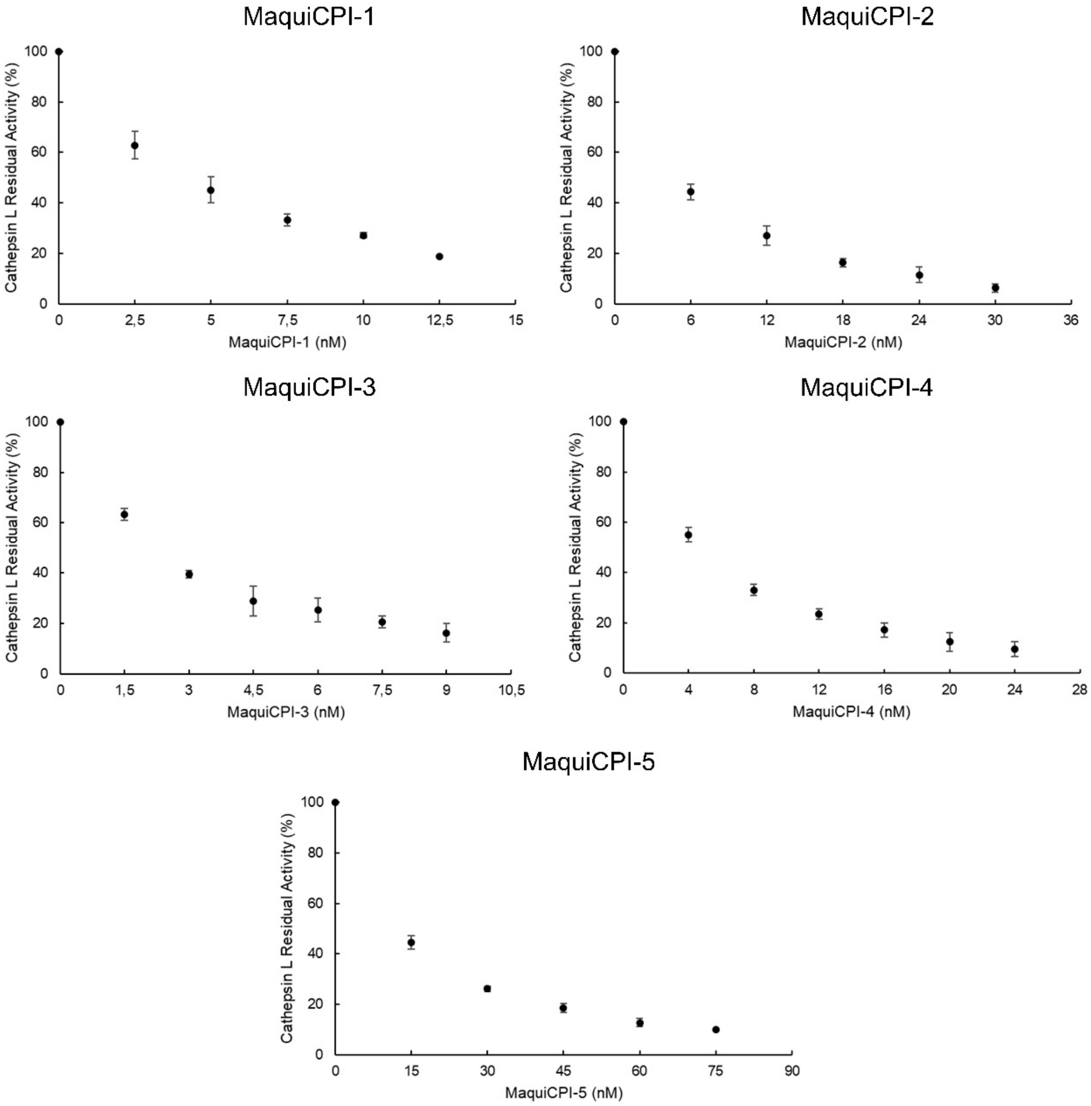

Figure S3: Human Cathepsin L inhibition by recombinant maquicystatins. The inhibitory activity of maquiCPIs against Cathepsin L was measured by a fluorimetric assay using Z-FR-MCA as a substrate. The graphics indicate the inhibition kinetics of each maquicystatin, which was obtained with the enzyme residual activity while increasing concentrations of inhibitor were added to the system. The values were indicated as the mean value ± the standard error of three independent experiments.
